# Circulatory microRNAs and proinflammatory cytokines as predictors of lupus nephritis

**DOI:** 10.3389/fimmu.2024.1449296

**Published:** 2024-10-11

**Authors:** Dalia Saad ElFeky, Noha Mohamed Omar, Olfat Gamil Shaker, Walaa Abdelrahman, Tamer A. Gheita, Mona Gamal Nada

**Affiliations:** ^1^ Department of Medical Microbiology and Immunology, Faculty of Medicine, Cairo University, Cairo, Egypt; ^2^ Department of Medical Biochemistry and Molecular Biology, Faculty of Medicine, Cairo University, Cairo, Egypt; ^3^ Rheumatology Department, Faculty of Medicine, Cairo University, Cairo, Egypt

**Keywords:** SLE, LN, IL-12, IL-21, miR-124, miR-146a, miR-199a, miR-21

## Abstract

**Introduction:**

Lupus nephritis (LN) is one of the most prevalent severe organ manifestations of systemic lupus erythematosus (SLE), impacting 70% of SLE patients. MicroRNAs (miRNAs), are small non-coding RNA molecules which influence the expression of approximately one-third of human genes after the process of transcription. Dysregulation of miRNAs was documented in numerous disorders, including SLE and LN. Cytokines are the orchestrators of the immune response in autoimmune diseases. Our study aims to explore the variation in the levels of circulating miRNAs and proinflammatory cytokines as potential diagnostic biomarkers among LN and SLE patients without LN in comparison to controls.

**Methods:**

The study involved 20 LN patients, 20 SLE patients without LN, and 10 healthy controls. Serum levels of IL-12 and IL-21 in addition to miR-124, miR-146a, miR-199a, and miR-21 were assessed using the enzyme-linked immunosorbent assay (ELISA) for cytokines and quantitative real-time PCR for miRNAs.

**Results:**

A significant downregulation in miR-124 (p<0.001) and a significant overexpression of miR-146a (p=0.005) were found in SLE patients without LN in comparison to controls. In comparison to SLE patients without LN and the control group, miR-199a, miR-21, and miR-146a were significantly upregulated in LN patients (p=<0.001) with high diagnostic values of these miRNAs in discriminating LN from SLE patients without LN according to Receiver operating curve (ROC) analysis. Logistic regression analysis revealed that only miR-199a is an independent predictor of LN (OR 1.69; 95% CI: 1.1-2.6). The expression of miR-124 was reduced in LN patients in comparison to the control but increased in LN patients in comparison to SLE patients without LN. However, there was no statistically significant difference in either scenario. In comparison to both SLE patients without LN and controls, LN patients exhibited the highest serum levels of IL-12 and IL-21, with no statistically significant difference. Regression analysis revealed that only miR-146a was associated with creatinine levels and SLEDAI score (p= 0.009 and 0.03, respectively), while miR-124 was associated with hemoglobin level (p=0.03).

**Conclusion:**

MiR-199a is an independent predictor for LN and might be used as a diagnostic biomarker for this disease. MiR-146a might play an important role in LN pathophysiology.

## 1 Introduction

Lupus nephritis (LN) is the most prevalent cause of death in patients with systemic lupus erythematosus (SLE), impacting as many as 70% of them (1). The percentage of people with LN developing end-stage renal disease (ESRD) ranges from 15% to 30%, depending on the degree of the disease severity (2). The pathogenesis of SLE and LN has been thoroughly studied, with significant research conducted on both genetic and environmental variables involved. Additionally, the significance of epigenetics in the development of these diseases has gained more recognition (3).

MicroRNAs (miRNAs) are non-protein-coding, single-stranded, 18–25 nucleotide RNA molecules that modify post-transcriptional gene expression. They have become recognized as potent regulators of several pathways and genes implicated in the pathophysiology of inflammatory illnesses in recent times (4, 5). Prior research has proved that there is an aberrant expression of miRNA in SLE and that it contributes to the progression of LN (6, 7). Advancing knowledge of the molecular, biological, and cellular characteristics of LN plays a crucial role in establishing diagnostic approaches and treatment techniques (8).

The established methods for clinically evaluating LN encompass conventional laboratory indicators, like complement and anti-double-stranded (ds) DNA levels, in addition to parameters related to renal involvement such as 24-hour proteinuria and glomerular filtration rate (GFR) (9). Although these methods are widely used to assess LN, they have shown poor effectiveness in rapid detection of LN flare and have limited accuracy in discriminating between active disease and long-term organ damage, which is crucial for efficient treatment planning of LN (10, 11).

Additionally, while kidney biopsy remains the primary method for confirming the diagnosis of LN and assess the degree and type of kidney damage (12), its invasive nature and the diverse causes of renal disease in SLE patients limit its utility in guiding treatment decisions (13). Fluid-based biomarkers, which are established indicators of physiological or pathological processes, or the treatment effectiveness, provide promising complementary less invasive alternatives for evaluating renal involvement in SLE (14, 15).

MiRNAs can be identified in both human cell lines and bodily fluids. Specific miRNA patterns in tissues and body fluids have been established to be indicative of certain disorders (16). Profiling of miRNAs that are expressed differentially provides valuable information on the epigenetic processes implicated in the pathogenesis of different diseases (17). This knowledge can potentially be used in practical fields for diagnosing and treating these disorders.

LN is more likely caused by the impact of several miRNAs rather than a single miRNA (18). Furthermore, several inflammatory pathways contribute to the emergence of end-organ symptoms in SLE, including LN. The type I interferon (IFN) and the nuclear factor kappa B (NF-κB) pathways play crucial roles in the inflammation processes associated with SLE and LN. miRNAs can modify these inflammatory pathways by regulating the innate and adaptive immune systems (19).

MiR-124 has a significant function in the regulation of both the innate and adaptive immune responses (20). It decreases the synthesis of interleukin (IL) 6 and tumor necrosis factor α (TNFα) by specifically targeting signal transducer and activator of transcription 3 (STAT3) and TNF-α’s converting enzyme (TACE) through the toll-like receptor (TLR) 4-induced cytokine pathway (21). Despite the importance of this miRNA, there is a lack of research specifically investigating its involvement in the progression of LN (21, 22).

MiR-146a is a negative regulator of innate signaling cascades. It is downregulated in SLE patients, which is linked to an increased type I IFN response (23). Despite its critical role, the data regarding miR146 expression in SLE and LN is contradictory, with some studies indicating its downregulation, while others report its elevation (19).

MiR-199a, which regulates inhibitory kappa B kinase-β (IKKβ) expression, is proved to be overexpressed and linked to the activation of NF-κB. This activation leads to an augmented synthesis of TNF-α and IL-1β in a human cell line model of LN (24). The latter study suggests that the patterns of miR-199 expression could serve as a novel diagnostic approach for predicting kidney involvement; however, they recommend further studies investigating its role in LN.

MiR-21 expression has been documented to be increased in CD4^+^ T-cells obtained from individuals with SLE and in lupus-prone MRL/lpr mice (25). While many studies have reported the upregulation of miR-21 in lupus patients in comparison healthy controls (3, 26–28), limited studies, to our knowledge, have examined its expression in LN patients (29).

Cytokines and chemokines are essential in the progression of LN, specifically in the recruitment of white blood cells and coordination of the inflammatory response (17).

Interleukin (IL)-12, in conjunction with IL-18, augments the production of IFN-γ and facilitates the proliferation or differentiation of naive T cells, acting as a strong inducer of TH1 cell differentiation (30). SLE patients were found to have increased levels of IL-12 in their blood (3, 31). Nevertheless, some studies have indicated a decrease in IL-12 expression in individuals diagnosed with SLE and LN (32, 33). Therefore, the exact contribution of IL-12 in the pathophysiology of LN is still uncertain, leading to a controversial understanding of its role (34).

Interleukin (IL)-21 is regarded as the primary cytokine that impacts the activation and differentiation of both T and B-cells (35). It is generated by CD 4^+^ T-cells, such as T helper 17 (TH17) and follicular CD4^+^ T-cells (36). IL-21 exhibits several effects that dictate the behavior of B cells in reaction to stimuli (37). It has been proved that IL-21 stimulates the secretion of IL-17, a key cytokine implicated in inflammatory processes like those seen in LN, thus prompting an inflammatory T-cell reaction (38). While numerous animal research have shown that IL-21 has a role in the pathophysiology of LN, there have been fewer investigations conducted on humans to understand this association (37, 39, 40).

While there has been considerable attention to the role of miRNAs in SLE development, fewer studies have specifically investigated their involvement in LN pathogenesis. Although the expression pattern of miRNAs in renal tissues may not be exactly the same as in the peripheral blood, the signaling transduction circuits implicated are clearly similar (24). Therefore, our work sought to further explore the pattern of expression of circulatory miRNAs and proinflammatory cytokines in SLE, with a specific focus on LN. Screening for miRNAs and cytokines could be beneficial in identifying predictive biomarkers and giving insights into their role in these diseases. Additionally, these biomolecules could be potential treatment targets for future studies.

## 2 Patients and methods

### 2.1 Participants enrollment

This study has been approved by the Research Ethical Committee, Faculty of Medicine, Cairo University (N-281-2023). It is a case-control design that included 40 patients diagnosed with SLE who were aged 18 or older and satisfied the revised criteria for SLE classification according to Aringer et al. (41). Additionally, ten healthy individuals who had the same age and gender as the SLE patients were included as controls. SLE patients involved 2 groups; 20 SLE patients diagnosed with LN and 20 SLE patients without nephritis. LN patients were diagnosed as having nephritis based on recommendations set by the American College of Rheumatology (42). Subjects were enrolled from the outpatient clinic at the Department of Rheumatology, Faculty of Medicine, Cairo University, between October 2023 and December 2023. Explicit consent was acquired from all participants in the study. Data on age, gender, clinical features, medications, and systemic lupus erythematosus activity index (SLEDAI) scores (43) were collected. The renal Systemic Lupus Erythematosus Disease Activity Index (rSLEDAI) was utilized to assess the activity of kidney disease, considering the presence of hematuria, pyuria, proteinuria, and urinary casts. Patients with a rSLEDAI score of 4 or higher were designated as having LN. Blood samples (3 mL) were obtained from both patients and controls. The serum was obtained from freshly collected blood samples by centrifuging them at a speed of 4000 times the force of gravity (4000 xg) for 10 minutes. The extracted serum was subsequently preserved at a temperature of -80°C until it was needed for further testing.

### 2.2 Measurement of serum level of IL-12 and 21

Serum levels of IL-12 and IL-21 were measured in sera of all participants using Human Interleukin 12 and 21 ELISA kits, Bioassay Technology Laboratory, Zhejiang, China (Cat. No E0099Hu and Cat. No E0057Hu, respectively) according to the manufacture’s protocol.

### 2.3 Measurement of serum levels of miRNAs expression

The RNA was obtained from serum using the miRNeasy mini kit (Qiagen, Valencia, CA, USA) and the purification process specifically intended for extracting total RNA from serum, which includes noncoding RNAs following manufacturer’s protocol. The RNA samples underwent RNA quantification and purity evaluation using the NanoDrop^®^ (ND)-1000 spectrophotometer (NanoDrop Technologies, Inc. Wilmington, USA). The miRNA analysis involved the reverse transcription (RT) of miRNAs into complementary DNAs (cDNAs) using the miScript II RT kit (Qiagen, Valencia, CA, USA). This procedure was performed on total RNA, following the manufacturer’s protocol, in a final volume of 20 uL. The miScript SYBR^®^ Green PCR kit (Qiagen, Valencia, CA, USA) was used to perform Quantitative Real-time PCR (qPCR) in a total volume of 25μL (**Table 1**). The qPCR was performed according to the manufacturer’s protocol, using the following primers: miR21 (Catalog no. YP00204230), miR-146a (Cat No MS00003535), miR-199a (Cat No. YP00204536) provided by Qiagen, Valencia, CA, USA, and Hsa-miR 124 (cat No MI0000443) provided by Thermofisher Scientific, USA. The Rotor-gene thermocycler (Qiagen, USA) was programmed as follows: an initial activation stage of PCR at 95°C for 15 minutes, followed by 40 cycles of denaturation at 94°C for 15 seconds, annealing at 55°C for 30 seconds, and extension at 70°C for 30 seconds.

**Table 1:** The reaction mix.

Component	Volume/reaction
2x QuantiTect SYBR Green PCR Master Mix	12.5 µl
10x miScript Universal Primer	2.5 µl
10x miScript Primer Assay *	2.5 µl
RNase-free water	Variable
Template cDNA (added at step 3)	≤ 2.5 µl
**Total volume**	25 µl

*The miscript primer assay: (miR-21, miR-124, miR-146a, miR-199a and SNORD 68).

#### 2.3.1 Results calculation

After completing the PCR cycles, melting curve analyses were used to confirm the accurate generation of the expected PCR product. Since there is a dearth of endogenous reference housekeeping genes for miRNA in serum, the SNORD 68 gene was utilized as a reference housekeeping gene to standardize the expression pattern and measure the target miRNAs. The ΔCt method was employed to assess the levels of expression of the target miRNAs. The cycle threshold (Ct) value represents the number of qPCR cycles required for the fluorescent signal to reach a predefined threshold. ΔCt was calculated by subtracting the Ct values of reference genes from those of the miRNAs of interest. ΔΔCt was calculated by subtracting the ΔCt value of control samples from that of disease samples. The fold change (FC) in miRNA expression was calculated using the equation 2–ΔΔCt.as follows:


$$\text{Δ}Ct_{\left(SLE*\;/\;LN\;patients\right)}=\;Ct_{\left(miRNA\right)}-\;Ct_{\left(Endogenous\;control\right)}$$

$$\text{Δ}Ct_{\left(Control\right)}=\;Ct_{\left(miRNA\right)}-\;Ct_{\left(Endogenous\;control\right)}$$

$$\text{ΔΔ}Ct_{\left(SLE\;/LN\;patients\right)}=\;\text{Δ}Ct_{\left(\text{SLE}/\text{LN patients}\right)}-\;\text{Δ}Ct_{\left(Control\right)}$$

$$FC\left(Rq\right)=2^{-\text{ΔΔ}Ct}$$

*SLE patients refer to SLE patients without LN.

**Figure 1:**
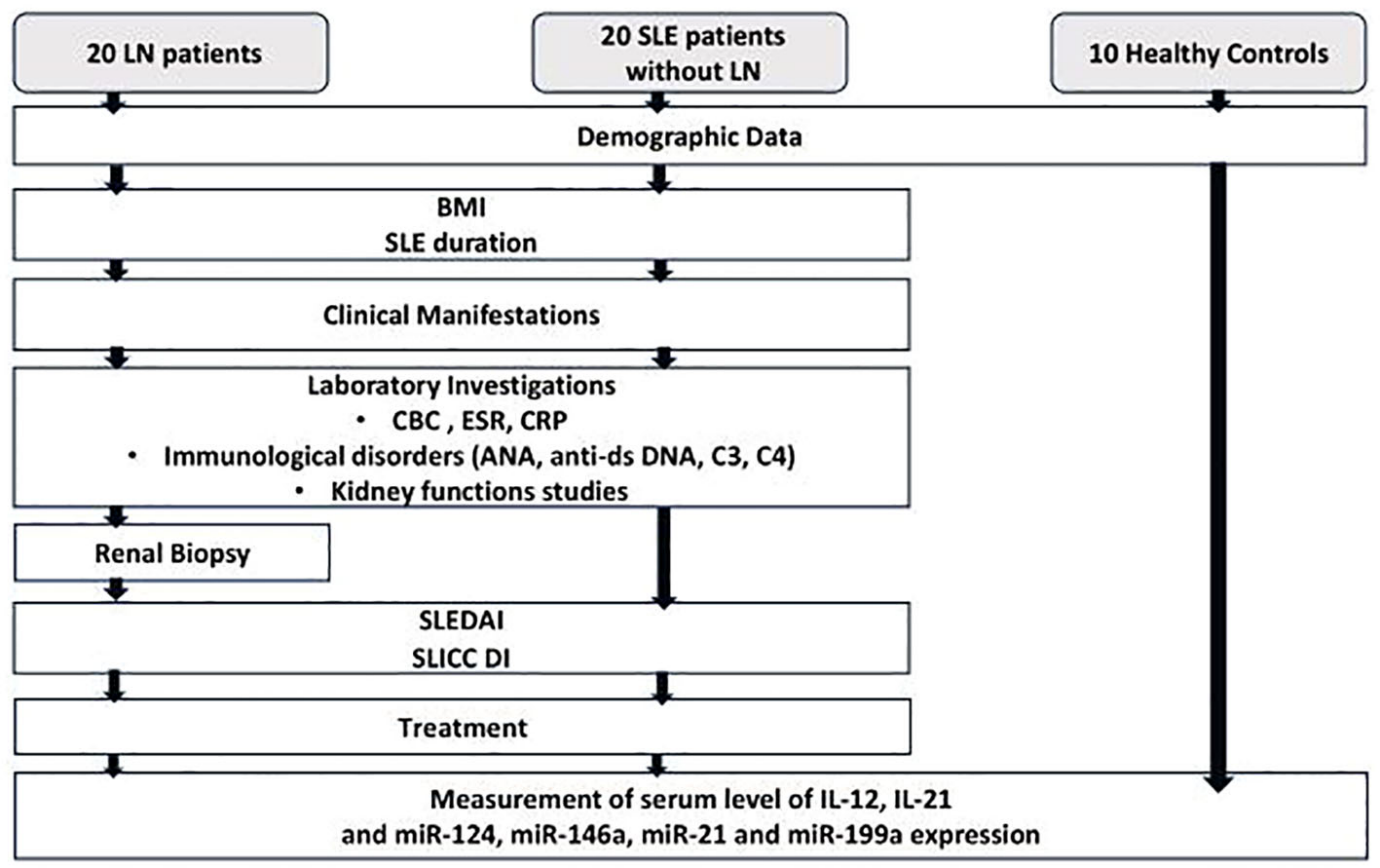
Flow chart for the study protocol. SLE, systemic lupus erythematosus; LN, lupus nephritis; BMI, body mass index; CBC, complete blood count; ESR, erythrocyte sedimentation rate; CRP, c-reactive protein; ANA, anti-nuclear antibody; Anti-dsDNA, anti-double stranded deoxyribonucleic acid; SLEDAI, systemic lupus erythematosus activity index; SLICC DI, Systemic lupus international collaborating clinics damage index; IL-12, Interleukin-12; IL-21, Interleukin-21; miR-124, micro RNA-124; miR-146a, micro RNA-146a; miR-21, micro RNA-21; miR-199a, micro RNA-199a.

A positive FC indicates upregulation of the miRNA, while a negative FC indicates downregulation. The control value was assigned as 1, as –ΔΔ*Ct* for control subjects is zero, and 2^0^ equals one. Schematic presentation of the study protocol is shown in **Figure 1**.

### 2.4 Statistical analysis

The data was encoded and analyzed using IBM Corp.’s (Armonk, NY, USA) statistical software package for the social sciences (SPSS) version 28. Quantitative data were described by mean, standard deviation and median, while count and percentage were used for categorical data. To compare quantitative and categorical data, respectively, the non-parametric Kruskal-Wallis and Mann-Whitney tests as well as the chi-square test were used (44). The exact test was used if the expected frequency is less than 5 (45). The associations between the clinical results and the miRNAs and cytokines under investigation were analyzed using the Spearman correlation (46) and linear regression analysis (47). To find out if distinct miRNAs function as independent predictors of LN, logistic regression was used (48). P-values were deemed statistically significant if they were less than 0.05. To precisely identify SLE and LN, a Receiver Operating Characteristic (ROC) curve was made, and an area under the curve (AUC) analysis was carried out.

## 3 Results

### 3.1 Characteristics of SLE patients

Our study investigated 40 SLE patients including 20 patients diagnosed with LN together with 10 healthy controls. Most of the patients were females (n=36) with only 4 males with a mean age of 33.4 ± 8.1 years. Patients were matched with the control group in terms of age (31.3 ± 7.9 years) and gender (8 females and 2 males), with no significant difference observed (p=0.47 and p=0.5 respectively).

Characteristics of SLE patients with and without LN are demonstrated in **Table 2**. The patients in the LN group developed nephritis after a mean of 6.7 ± 5.04 years and their mean proteinuria was 2.04 ± 2.62 g/24 hr. Patients with LN had a considerably greater occurrence of hypertension, dyslipidemia, and metabolic syndrome compared to those without LN. All patients tested positive for ANA, with 50% (n= 10) showing a speckled pattern, 45% (n=9) showing a homogeneous pattern, and 5% (n=1) showing a rim pattern in those with LN. In SLE patients without LN, 75% (n=15) showed a speckled pattern and 25% (n=5) showed a homogenous pattern. The frequency of anti-dsDNA was substantially greater in patients with LN compared to those without. Patients with LN showed an increased consumption rate of C3 and C4, although this disparity did not reach statistical significance. In addition, the patients in the nephritis group had a significantly greater SLEDAI score. Statistically significant greater doses of steroids were administered to nephritis patients compared to other patients. Only patients diagnosed with LN were being administered cyclophosphamide. Additionally, a higher proportion of patients with LN were receiving mycophenolate mofetil in comparison to those without LN, and this difference was statistically significant.

**Table 2:** Characteristics of the SLE patients with and without LN.

Variable Mean ± SD or n (%)	SLE patients (n=40)		*p-value*
	LN (n=20)	without LN (n=20)	
Age (years)	32.5 ± 7.9	34.3 ± 8.5	0.5
Gender F:M	17:03	19:01	0.31
Age at onset (years)	24.9 ± 7.3	28.2 ± 8	0.18
SLE duration (years)	8.7 ± 5.1	6.1 ± 5.8	0.14
BMI	28.8 ± 5.2	29.7 ± 7	0.82
Diabetes	3 (15)	1 (5)	0.31
Hypertension	15 (75)	3 (15)	<**0.0001**
Dyslipidemia	19 (95)	13 (65)	**0.019**
Metabolic syndrome	12 (60)	4 (20)	**0.009**
Constitutional	15 (75)	17 (85)	0.44
Mucocutaneous	20 (100)	16 (80)	**0.04**
Musculoskeletal	17 (85)	18 (90)	0.64
Pulmonary	4 (20)	6 (30)	0.48
Cardiac	5 (25)	3 (15)	0.44
CNS	5 (25)	5 (15)	1
PNS	0 (0)	1 (5)	–
Hematologic	10 (50)	12 (60)	0.54
GIT	1 (5)	3 (15)	0.31
Vasculitis	1 (5)	3 (15)	0.31
Ocular	0 (0)	3 (15)	–
Thrombosis	2 (10)	5 (15)	0.22
APS	4 (20)	8 (40)	0.21
ESR (mm/1^st^ hr)	44.9 ± 13.3	36.3 ± 24.3	0.18
CRP positive	2 (10)	2 (10)	1
Hemoglobin (g/dl)	11.2 ± 2	11.6 ± 1.4	0.46
TLC (x10^3^/mm^3^)	7.1 ± 2.4	6 ± 2.2	0.14
Platelets (x10^3^/mm^3^)	258.6 ± 82.2	235.1 ± 100.9	0.43
Creatinine (mg/dl)	1.03 ± 0.4	0.71 ± 0.14	**0.003**
Urea (mg/dl)	42.1 ± 27.1	29.5 ± 9.7	0.06
s.Albumin (mg/dl)	3.5 ± 0.99	4 ± 0.43	0.06
SUA (mg/dl)	5.7 ± 1.7	4.4 ± 1.7	**0.02**
Current proteinuria	13	0	**0.001**
Hematuria	1	1	1
Pyuria	6	0	**0.02**
Casts	0	1	1
GFR (ml/min/1.73m^2)	81.6 ± 33.4	100.4 ± 29.6	0.07
Consumed C3	6 (30)	2 (10)	0.14
Consumed C4	4 (20)	1 (5)	0.17
ANA	20 (100)	20 (100)	1
Anti-dsDNA	16 (80)	10 (50)	**0.047**
SLEDAI	3.2 ± 2.86	0.4 ± 0.99	**<0.0001**
Remission	13 (65)	4 (20)	**0.003**
SLICC DI	0.55 ± 1	0.5 ± 0.76	0.86
Steroids (mg/d)	13.5 ± 4.4	8.9 ± 4.1	**0.001**
Cyclophosphamide	10 (50)	0 (0)	–
Hydroxychloroquine	14 (70)	13 (65)	0.74
Azathioprine	3 (15)	6 (30)	0.27
Mycophenolate mofetil	14 (70)	6 (30)	**0.01**
Cyclosporin A	2 (10)	1 (5)	0.56
Rituximab	0 (0)	4 (20)	–

SD, standard deviation; SLE, systemic lupus erythematosus; LN, lupus nephritis; BMI, body mass index; ESR, erythrocyte sedimentation rate; CRP, c-reactive protein; TLC, total leucocytic count, GFR, glomerular filtration rate; ANA, anti-nuclear antibody; Anti-dsDNA, anti-double stranded deoxyribonucleic acid; SLEDAI, systemic lupus erythematosus activity index; SLICC DI, Systemic lupus international collaborating clinics damage index. Bold values are significant at p<0.05.

### 3.2 Renal biopsy findings in LN patients

Out of the 20 LN cases, nephritis was definitively diagnosed through renal biopsy in 15 patients (75%). In the other 5 LN patients, the diagnosis was made according to the recommendations set by the American College of Rheumatology (42). Renal biopsy findings are presented in **Table 3**. Class IV was the most predominant among the cohort (50%). Their mean activity index was 6.55 ± 5.36 and their mean chronicity index was 1.5 ± 1.6.

**Table 3:** Biopsy findings in patients with LN.

Renal biopsy findings	LN (n=20)
Class: 0 (No biopsy)	5 (25)
III	4 (20)
IV	10 (50)
IV/V	1 (5)
Activity index	6.55 ± 5.36
Endocapillary Hypercellularity	1.55 ± 1.28
Leukocyte Infiltration	1.45 ± 1.28
Subendothelial Hyaline Deposits	0.5 ± 1.1
Fibrinoid Necrosis	0.45 ± 1
Cellular Crescents	0.4 ± 0.82
Interstitial Inflammation	1.55 ± 1.43
Chronicity Index	1.5 ± 1.61
Glomerular Sclerosis	0.9 ± 0.72
Fibrous Crescents	0
Tubular Atrophy	0.15 ± 0.67
Interstitial Fibrosis	0.45 ± 0.83

### 3.3 Serum levels of ILs and fold change in expression of miRNAs in different groups

**Figure 2:**
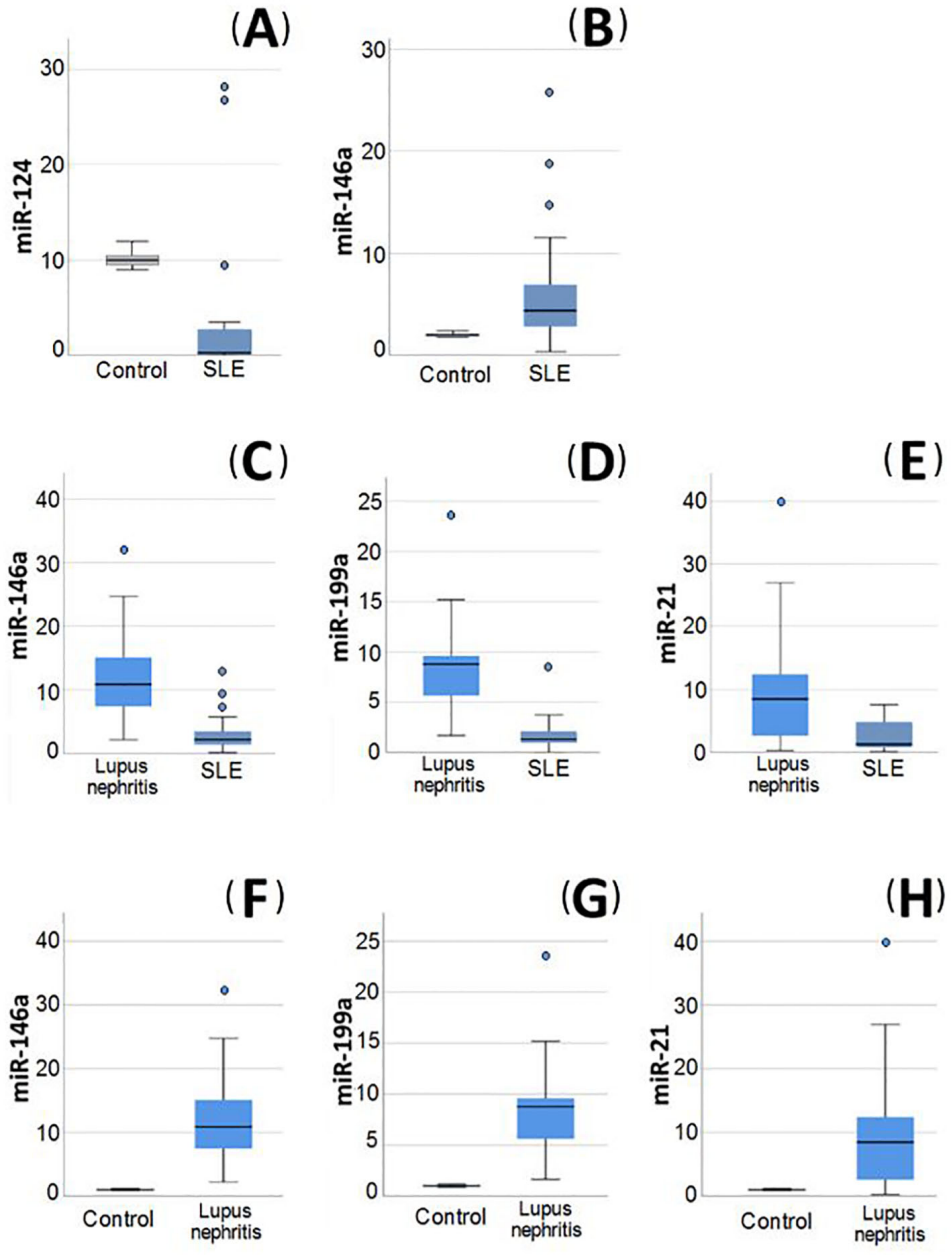
Relative expression of miRNAs in blood of the studied groups. **(A, B)** Relative expression of miRNAs in systemic lupus erythematosus (SLE) without lupus nephritis (LN) patients compared to healthy control, **(A)** miR-124 was significantly decreased in SLE patients without LN compared to healthy control. **(B)** miR-146a was significantly increased in SLE patients without LN compared to healthy control. **(C–E)** Relative expression of miRNAs in LN patients compared to SLE without LN patients. **(C)** miR-146a was significantly increased in LN patients compared to SLE patients without LN. **(D)** miR-199a was significantly increased in LN patients compared to SLE patients without LN. **(E)** miR-21 was significantly increased in LN patients compared to SLE patients without LN. **(F–H)** Relative expression of miRNAs in LN patients compared to control group. **(F)** miR-146a was significantly increased in LN patients compared to controls. **(G)** miR-199a was significantly increased in LN patients compared to controls. **(H)** miR-21 was significantly higher in LN patients compared to controls.

Serum levels of IL-12, IL-21, and FC in miR-124, miR-146a, miR-199a, and miR-21 expression in SLE patients with and without LN as well as control groups are presented in **Table 4**. On comparing the FC between patients versus control, it was evident that miR-124 exhibited a statistically significant decrease among SLE patients without LN, while miR-146a was substantially upregulated (p= <0.001 and 0.005, respectively) (**Figures 2A, B**). Among the patients in the same group, there was a rise in the IL-12, IL-21 levels, and FC of miR-199a and miR-21 in their serum in comparison to the control group. However, this discrepancy did not achieve statistical significance.

**Table 4:** Comparison between serum level of IL-12, IL-21and fold change (FC) of miR-124, miR-146a, miR-21 and miR-199a expression in the studied groups.

Serum level/Fold change (FC) (mean ± SD)	SLE patients (n=40)		Control (n=10)	*P-value*		
	LN (n=20)	Without LN (n=20)		P1	P2	P3
IL-12	214.5 ± 189.7	154.02 ± 143.4	112.5 ± 33.3	0.448	0.681	0.134
IL-21	574.5 ± 397.9	531.5 ± 419.02	420.6 ± 264.2	0.422	0.948	0.398
MiR-124	0.59 ± 0.72	0.39 ± 0.84	1.02 ± 0.09	0.06	**< 0.001**	0.06
MiR-146a	12.3 ± 7.5	3.4 ± 3.2	1.03 ± 0.1	**< 0.001**	**0.005**	**< 0.001**
MiR-21	10.4 ± 10.04	2.5 ± 2.5	1.03 ± 0.1	**< 0.001**	0.328	**< 0.001**
MiR-199a	8.7 ± 4.9	1.8 ± 1.8	1.03 ± 0.1	**< 0.001**	0.248	**< 0.001**

SD, standard deviation; SLE, systemic lupus erythematosus; miR, micro ribonucleic acid; LN, lupus nephritis; P1, between LN and control; P2, between SLE without LN and control; P3, between SLE with and without LN. Bold values are significant at p<0.05.

In LN patients, miR-199a, miR-146a, and miR-21 were shown to be significantly elevated (p= <0.001) in comparison to both SLE patients without LN (**Figures 2C–E**) and the control group (**Figures 2F–H**). The miR-199a showed the greatest FC overexpression (4.8), followed by miR-21 (4.16) and finally miR-146a (3.6). Additionally, logistic regression analysis revealed that only miR-199a is an independent predictor of LN (OR 1.69; 95% CI: 1.1-2.6).

The expression of miR-124 was shown to be reduced in LN patients in comparison to the control group, nevertheless, this difference was not statistically significant. Interestingly, the levels of miR-124 were greater in LN patients in comparison to SLE patients without LN, but this difference was not statistically significant.

Concerning the studied parameters in all patients, only serum level of IL-21 was significantly higher in females (561.9 ± 391.5) compared to males (267.01 ± 127.5; p=0.001). Patients with metabolic syndrome (n=16) had a significantly higher FC increase in miR-199a (7.41 ± 5.83) compared to those without (3.8 ± 3.9; 0.038). Additionally, in patients with hypertension (n=18), there was a significant elevation in FC of miR-146a, mi-R21 and miR-199a (p=0.01, p=0.02 and p=0.001). Cases with dyslipidemia (n=32) had a significantly increased IL-12 levels (p=0.002) and increase FC of miR-124 (p<0.0001) compared to those without.

Patients with pulmonary manifestations (n=10) had a significantly less fold increase of miR-146a (4 ± 3.5) compared to those without (9.1 ± 7.8; p=0.008) also those with GIT involvement (n=4) had a significantly lower fold increase of miR-21 (2.2 ± 2.8) vs those without (7 ± 8.5; p=0.037). Those with vasculitis (n=4) had significantly reduced IL-21 levels and FC of miR-124 (253 ± 147.4 and 0.04 ± 0.05 vs 586.3 ± 410.5 and 0.54 ± 0.8; p=0.008 and p=0.001, respectively). Cases with thrombosis (n=7) had a significantly lower level of IL-12 and lower FC of miR-124 (102.2 ± 32.7 and 0.13 ± 0.12 vs 201.7 ± 180.9 and 0.57 ± 0.84; p=0.006 and p=0.007 respectively). Levels of the studied parameters were comparable between those with and without other manifestations. IL-12 and FC in miR-146a expression were significantly increased in those with disease remission (n=17) (269.02 ± 200.1 and 11.3 ± 8.3 vs 121.6 ± 108.3 and 5.3 ± 5.2; p=0.01 and p=0.008 respectively). Regarding the laboratory findings, only IL-12 was significantly decreased in those with consumed C3 (n=8) (108.6 ± 30.2 vs 207 ± 185.6; p=0.008). The levels of the studied parameters were similar between those receiving medications and those not.

### 3.4 Correlation between IL-12, IL21 and the studied miRNAs

As shown in **Table 5**, correlation analysis revealed a substantial association between IL-12 and IL-21 (r=0.6, p<0.0001). On the other hand, the IL-21 showed a significant correlation with miR-124 (r=0.39, p=0.012). The miRNA- 124 also showed a notable association with miR-199a (r=0.47, p=0.002). The miR-146a was significantly correlated with both miR-199a (r=0.64, p<0.0001) and miR-21 (r=0.44, p=0.005). Additionally, there was a statistically significant correlation between miR-199a and miR-21 (r=0.51, p=0.001).

**Table 5:** Correlations between ILs and miRNAs.

	IL-12 (ng/L)	IL-21 (ng/L)	miR-124	miR-146a	miR-21	miR-199a
	r (p-value)					
**IL-12**	1.000 (-)	0.6 (**<0.0001)**	0.292 (0.068)	0.185 (0.253)	0.026 (0.873)	0.265 (0.098)
**IL-21**	0.6 (**<0.0001)**	1.000 (-)	0.394 (**0.012)**	0.168 (0.300)	-0.06- (0.737)	0.140 (0.388)
**miR-124**	0.292 (0.068)	0.394 (**0.012)**	1.000	0.135 (0.408)	0.167 (0.304)	0.469 (**0.002)**
**miR-146a**	0.185 (0.253)	0.168 (0.300)	0.135 (0.408)	1.000 (-)	0.436 (**0.005)**	0.64 (**< 0.001)**
**miR-21**	0.026 (0.873)	-0.06- (0.737)	0.167 (0.304)	0.436 (**0.005)**	1.000 (.)	0.512 (**0.001)**
**miR-199a**	0.265 (0.098)	0.14 (0.388)	0.469 (**0.002)**	0.638 (**<0.001)**	0.512 (**0.001)**	1.000 (-)

r*, Correlation coefficient; Bold values are significant at p<0.05.

### 3.5 Correlation between the studied parameters and clinical parameters of LN and biopsy findings

The correlation of the studied parameters with some features, laboratory investigations, disease activity and damage as well as drug doses are presented in **Table 6**. The expression of miR-124 showed a positive correlation with the creatinine level (r= 0.33, p= 0.04), but a negative correlation with the hemoglobin level (r=-0.04, p= 0.005) and activity index (r= -0.7, p= 0.002).A significant negative association was observed between miR-146a and both age and age at the onset of SLE (r= -0.36, p= 0.02; r= 0.44, p= 0.01, respectively). Moreover, there was significant positive correlation between miR-146a and creatinine level, steroid dose, proteinuria and SLEDAI score (r= 0.32, p= 0.04; r= 0.4, p= 0.008; r= 0.36, p= 0.02; r=0.44, p= 0.004, respectively). MiR199a was significantly correlated to SLE duration, urea level, creatinine level, proteinuria and SLEDAI score (r= 0.33, p= 0.04; r= 0.32, p= 0.04; r= 0.44, p= 0.005; r= 0.6, p= <0.001; r= 0.45, p= 0.004, respectively). A significant negative association was found between miR-199a and azathioprine dose (r= -0.65, p= 0.04). Whereas miR-21 was significantly correlated to nephritis duration and proteinuria (r= 0.47, p=0.04; r= 0.5, p= <0.001, respectively) and negatively correlated to C4 level (r= -0.44, p= 0.04). IL-12 was correlated only negatively to GFR (r= -0.3, p= 0.048), while IL-21 was correlated negatively to hemoglobin level (r= -0.35, p= 0.03) and positively to azathioprine dose (r= 0.81, p= 0.005). None of the studied candidates were correlated to renal biopsy class.

**Table 6:** Correlation between the studied parameters and clinical characteristics of LN.

	IL-12	IL-21	miR-124	miR-146a	miR-21	miR-199a
	r (p-value)					
**Age (n=40)**	0.14 (0.4)	-0.057- (0.73)	-0.01- (0.9)	-0.36- **(0.02)**	-0.14- (0.4)	-0.06- (0.7)
**age at onset of SLE (n=40)**	-0.03- (0.9)	-0.21- (0.2)	-0.11- (0.5)	-0.44- **(0.01)**	-0.3- (0.1)	-0.27- (0.1)
**SLE duration (n=40)**	0.23 (0.16)	0.24 (0.14)	0.19 (0.25)	0.25 (0.13)	0.18 (0.26)	0.33 **(0.04)**
**nephritis duration (n= 20)**	0.41 (0.07)	0.32 (0.17)	0.06 (0.81)	0.15 (0.54)	0.47 **(0.04)**	-0.06- (0.8)
**Hb (gm/dl) (n=40)**	-0.2- (0.22)	-0.35- **(0.03)**	-0.4**-(0.005)**	-0.07- (0.7)	-0.19- (0.2)	-0.24- (0.14)
**TLC (1000/cm3) (n=40)**	0.29 (0.07)	0.1 (0.53)	-0.1- (0.53)	0.2 (0.22)	0.07 (0.69)	0.17 (0.31)
**PLT count (1000/cm3) (n=40)**	-0.23- (0.16)	0.07 (0.66)	-0.08- (0.6)	0.09 (0.57)	0.18 (0.27)	-0.08- (0.62)
**CRP (mg/dl) (n=40)**	0.12 (0.47)	0.28 (0.09)	0.24 (0.13)	0.07 (0.67)	0.23 (0.15)	0.25 (0.12)
**24 Hrs Ptn (g/d) (n=40)**	0.09 (0.57)	-0.08- (0.62)	0.19 (0.23)	0.36 **(0.02)**	0.5 (**< 0.001)**	0.6 (**< 0.001)**
**urea (mg/dl) (n=40)**	0.01 (0.96)	-0.05-(0.77)	0.27 (0.09)	0.06 (0.71)	0.26 (0.11)	0.32 **(0.04)**
**creatinine (mg/dl) (n=40)**	0.26 (0.1)	0.14 (0.4)	0.33 (**0.04)**	0.32 **(0.04)**	0.14 (0.38)	0.44 **(0.005)**
**GFR (ml/min/1.73m^2) (n=40)**	-0.3-**(0.048)**	-0.17-(0.27)	-0.23-(0.2)	-0.18-(0.26)	0.02 (0.91)	-0.28-(0.08)
**C3 (mg/dl) (n=24)**	0.15 (0.49)	0.29 (0.18)	0.15 (0.5)	-0.06- (0.78)	-0.29- (0.17)	-0.1- (0.65)
**C4 (mg/dl) (n=22)**	-0.13- (0.58)	0.08 (0.72)	0.34 (0.12)	-0.16- (0.49)	-0.44- **(0.04)**	-0.25- (0.25)
**SLEDAI (n=40)**	0.29 (0.07)	0.13 (0.42)	0.27 (0.09)	0.44 **(0.004)**	0.13 (0.41)	0.45 **(0.004)**
**Activity index (n=15)**	-0.03- (0.9)	-0.08- (0.77)	-0.7-**(0.00)**	-0.41- (0.12)	-0.11- (0.6)	-0.27- (0.33)
**Chronicity index (n=15)**	0.17 (0.54)	-0.1- (0.7)	0.1 (0.73)	-0.08- (0.78)	0.09 (0.74)	-0.11- (0.7)
**Class (n=15)**	-0.29- (0.3)	-0.39- (0.15)	-0.38- (0.2)	-0.27-(0.34)	0.12 (0.7)	-0.16- (0.58)
**steroids current dose (mg) (n=40)**	0.18 (0.26)	0.27 (0.09)	0.09 (0.57)	0.4 (**0.008)**	0.15 (0.36)	0.24 (0.14)
**AZA dose (mg) (n=10)**	0.54 (0.11)	0.81 **(0.005)**	0.11 (0.76)	-0.34- (0.34)	-0.63- (0.05)	-0.65-**(0.04)**
**MMFdose (gm) (n=24)**	-0.13- (0.55)	-0.12- (0.58)	0.3 (0.16)	0.3 (0.15)	0.17 (0.44)	0.4 (0.5)
**BMI (n=40)**	0.16 (0.32)	0.22 (0.17)	0.03 (0.87)	0.007 (0.97)	0.007 (0.96)	0.13 (0.44)

r, correlation coefficient; Hb, hemoglobin; TLC, total leucocytic count; PLT, platelets; CRP, C-reactive protein, 24-hr-ptn, 24-hour proteinuria; GFR, glomerular filtration rate; SLEDAI, systemic lupus erythematosus disease activity index; AZA, azathioprine; MMF, mycophenolate mofetil; BMI, body mass index; Bold values are significant at p<0.05.

**Figure 3:**
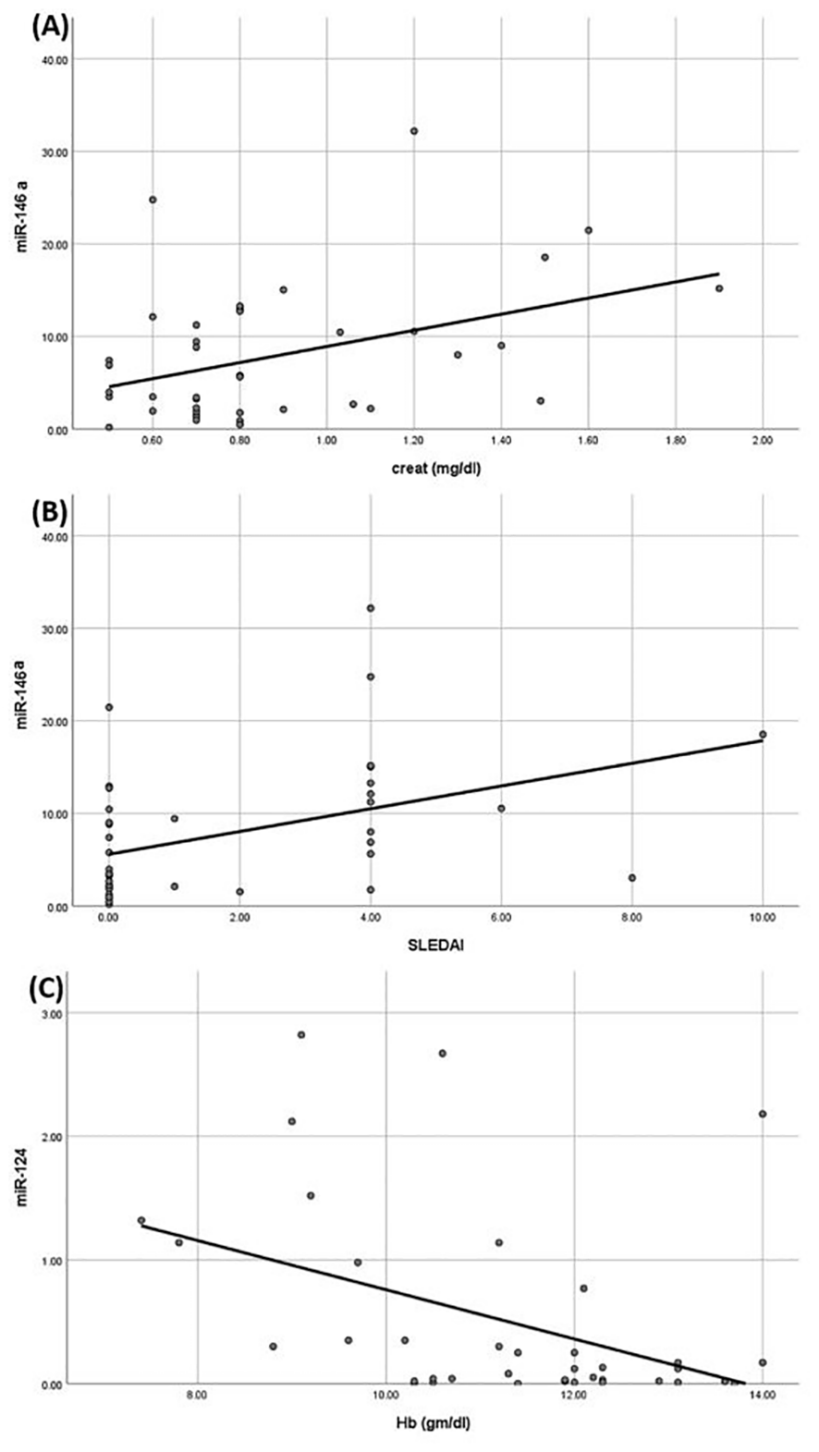
Linear regression analysis between miRNAs understudy and clinical parameters. **(A)** Positive correlation between miR-146a and creatinine. **(B)** Positive correlation between miR-146a and systemic lupus erythematosus activity index (SLEDAI) score. **(C)** Negative correlation between miR-124 and hemoglobin.

On regression analysis of all the correlated parameters with studied miRNAs and cytokines, none remained significant. Only the association of serum creatinine and SLEDAI with miR146 remained significant (p= 0.009 and 0.03, respectively) and the association of Hb with miR124 (p=0.03) (**Figure 3**).

### 3.6 The diagnostic value of the studied parameters on SLE and LN

**Figure 4:**
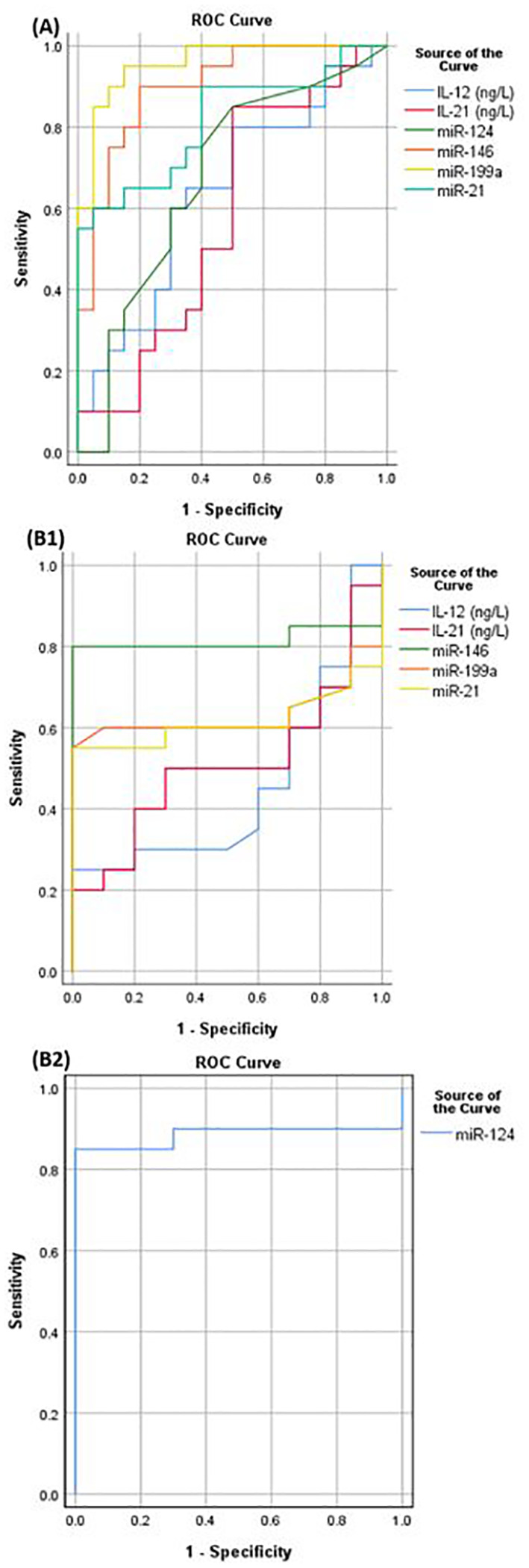
**(A)** ROC curve to discriminate between LN and SLE patients without LN, **(B1)** ROC curve to differentiate between SLE patients without LN and control group, **(B2)** ROC curve to test miR-124 ability to discriminate between SLE patients without LN and control.

A ROC analysis was constructed in order to evaluate the diagnostic efficacy of the parameters under investigation in discriminating between SLE patients with and without LN as depicted in **Figure 4A**. It was found that miR-146a would significantly differentiate with area under the curve (AUC) 0.9 at cutoff value 3.7 (sensitivity 90%, specificity 80%, p= <0.001). Similarly, miR-21 demonstrated discriminatory power with a cutoff 7.72 and AUC of 0.82 (sensitivity 55%, specificity 100%, p= <0.001). Additionally, miR-199a displayed a significant discriminatory ability with a cutoff of 3.9 and an AUC of 0.96 (sensitivity 85%, specificity 95%, p<0.0001) and miR-124 with a cutoff of 0.05 and an AUC of 0.68 (sensitivity 80%, specificity 55%, p=0.046). However, the studied interleukins could not significantly discriminate between SLE patients with and without LN.

Furthermore, a ROC analysis was constructed to assess the diagnostic efficacy of the studied parameters in distinguishing SLE patients without LN from the control group, as illustrated in **Figure 4B1**. It was found that miR-146a would significantly differentiate with area under the curve 0.82 at cut off value 1.24 (sensitivity 80%, specificity 100%, p<0.0001). Similarly, miR-124 exhibited substantial discriminatory power with a cutoff of 0.63 and an AUC of 0.89 (sensitivity 85%, specificity 100%, p=<0.0001) as shown in **Figure 4B2**. However, the other parameters could not significantly differentiate between SLE patients without LN and the control.

## 4 Discussion

LN is one of the most devastating conditions linked to SLE. It is typically associated with a poor outcome, with one third of LN patients eventually developing end-stage renal failure (49). It is crucial to find biomarkers that are noninvasive to improve our comprehension of disease pathogenesis, enable early diagnosis and the potentiality of being a therapeutic target for LN treatment (19). While numerous research investigations have emphasized the participation of miRNAs in the development of SLE, fewer have delved into their role specifically in LN pathogenesis. Therefore, the purpose of this work was to conduct additional research on the function of miRNAs in SLE, with a specific focus on LN, and to explore their relationship with pro-inflammatory cytokines.

In our study, we investigated miR-124, one of the known negative regulators of inflammation whose level has been reported to be downregulated in some autoimmune diseases such as rheumatoid arthritis and SLE (50). Our investigation demonstrated a significant reduction in miR-124 expression levels in SLE patients in comparison to controls. This finding further supports the existing evidence suggesting that miR-124 plays a protective role in SLE. This observation aligns with previous research (51, 52). Conversely, a prior study reported an increase in miR-124-3p expression in individuals with SLE (53). The researchers claimed that it impacts SLE by suppressing the transcription of early growth response-1 (EGR1), a protein that controls the attachment and survival of healthy cells, which is reduced in SLE (54, 55). Nevertheless, this work specifically examined miR-124-3p, which is derived from the 3′ end arm of the miR-124 precursor.

Additionally, we found that miR-124 was downregulated in LN patients than in non-diseased control group, but the discrepancy was not statistically significant. However, other studies demonstrated a significant downregulation of miR-124 in LN patients in comparison to healthy controls (21, 22). Interestingly, we noticed that miR-124 levels were higher in LN group in comparison to SLE patients without LN, but this difference did not achieve statistical significance. This finding contrasts with Zhang et al. (22), who observed significantly lower miR-124 expression in LN patients compared to SLE patients without LN. They found that miR-124 overexpression, by downregulating the target gene TNF-receptor associated factor 6 (TRAF6), inhibits cell proliferation and decreases the production of inflammatory factors in human renal mesangial cells. The different observed results in our study could be potentially attributed to factors such as the small sample size, genetic variations, or the influence of extensive immunosuppressive therapy received by these patients.

Another miRNA of interest is miR-146a which is known to regulate the inflammatory response by targeting TLRs and other effectors like TRAF6, thus preventing the transcription of NF-κB (56). Despite its crucial role, the data about miR146 expression in SLE is controversial. Some studies including those by Singh et al. (3) and Zhu et al. (57) have observed that miR-146 is downregulated in SLE and LN, respectively. A recent study in Egypt by Higazi et al. (58) stated that miR-146 was decreased in SLE patients with a tendency to be even lower in LN patients.

However, our investigation demonstrated that the levels of miR-146a were considerably elevated in both SLE patients with and without nephritis compared to the healthy controls. Furthermore, the expression of this miRNA was markedly higher in LN patients in comparison to those without nephritis. Consistent with our finding, previous studies reported a significant elevation of miR-146 in individuals with SLE compared to a group of healthy individuals (59–62). Previous studies similarly found a notable elevation in miR-146 levels in LN patients in comparison to healthy control (2, 63). Additionally, these results agree with Monticelli et al. (64) who observed that miRNA-146a expression is more prevalent in Th1 cells and less in Th2 cells, in murine model and is linked with the expression of Th1-related proinflammatory cytokines genes such as TNFα and IFN-γ. Nevertheless, a study by Khoshmirsafa et al. (7) observed no statistically significant disparity in miR-146 levels between LN group and either SLE without LN group or healthy controls.

The discrepancy in miR146 expression might be a result of environmental factors, different treatment approaches and genetic predisposition as evidenced by Löfgren et al. (65) who observed a correlation between a specific single- nucleotide polymorphism (SNP) and miR-146a expression which might lead to upregulation of miR-146.

Moreover, we conducted an analysis of miR-199a expression levels and observed that they were elevated in individuals with SLE in comparison to the healthy control. However, the disparity did not achieve statistical significance. Significantly, miR-199a was upregulated in LN patients relative to both SLE patients without LN and the control group. The significant increase in expression, surpassing that of all other miRNAs examined in our study on blood samples from LN patients, indicates that miR-199a might play a significant function in the pathophysiology of LN.

These findings align with earlier research that found a significant upregulation of miR-199 expression in patients with SLE (66) and LN patients (18, 67) compared to healthy individuals. According to So et al. (19), there is evidence indicating that miR-199 levels were increased and correlated with the activation of the NF-kB pathway, suggesting its involvement in inflammation. In their research on LN patients, Ye et al. (68) discovered that Klotho, a membrane protein found in the human kidney, has antioxidant and reno-protective properties, negatively regulates NF-κB-associated inflammation in LN patients. Additionally, they observed that miR-199a downregulates the expression of Klotho *in vitro*. In contrast to our results, Elessawi et al. (24) reported downregulation of miR-199 expression LN patients relative to those without LN. This suggests that the expression of miRNAs may vary across various cohorts of patients.

As it has been established that miR-21 is related to many autoimmune disorders like psoriasis, multiple sclerosis and SLE, it was one of the studied miRNAs in our research. We have found that miR-21 was increased in SLE patients relative to the control group, although this disparity was not statistically significant. However, earlier research has consistently shown a significant increase in miR-21 expression among SLE patients in comparison to healthy controls (27, 59, 61). The dysregulation of miR-21 has been found to enhance the occurrence of SLE by directly and indirectly regulating CD4+ T cells (69). It was found that miR-21 regulates forkhead box P3 (FoxP3) expression positively and T-regs negatively (70).

In line with previous research which provided evidence that miR-21 might be associated with renal disease development (71), our findings demonstrated that it was markedly upregulated in LN patients in comparison to SLE patients without LN and the group of healthy control. In accordance with our finding, previous studies have demonstrated that miR-21 was significantly increased in LN patients on comparing to healthy control (7, 29) and to SLE patients without LN (2).

The representation of miRNA levels in the blood of individuals with SLE and LN varies greatly and sometimes contradicts across studies. Factors such as cohort racial and ethnic composition, disease activity fluctuations, immunosuppressive treatments, and technical issues may contribute to this. High circulating RNase levels, known to reduce circulating miRNAs in advanced chronic kidney disease (CKD), make it challenging to compare and extrapolate results across different stages of CKD in LN patients and animal models (72).

We additionally explored whether the inflammatory cytokines IL-12 and IL-21 exhibited elevated levels in SLE patients and assessed their involvement in LN.Top of Form We have found upregulated protein levels of IL-12 in sera of SLE patients relative to the healthy control, but the disparity did not achieve statistical significance. Nevertheless, it has been documented that IL-12 levels are notably increased in SLE patients (3, 31, 73–75). The exact function of IL-12 in the development of LN is not well comprehended (34). We have noticed elevated levels of IL-12 in LN more than SLE patients without LN and control, although the disparity was not statistically significant. Few studies have researched IL-12’s role in LN pathogenesis. An immunohistochemical study on human renal tissues found that IL-12 was significantly higher among proliferative LN than control group (76). Moreover, Tucci et al. (77) determined that IL-12 triggers inflammation in the kidneys and promotes an imbalance of cytokines in peripheral cells, preferring a Th1 phenotype.

IL-21 indeed holds a substantial function in the development and functionality of T cells (38). Our findings showed elevated protein levels of IL21 in SLE patients, with a tendency for higher levels among the LN subgroup, although this discrepancy did not attain statistical significance possibly because of the limited number of participants in our study. Previous research has consistently observed a significant rise in IL-21 levels in SLE patients on comparing with controls (3, 38, 78). Additionally, Shater et al. (37) demonstrated a statistically significant elevation of IL-21 in active LN patients compared to both non-active LN patients and the controls. A previous study discovered that in a lupus susceptible mouse model, elevated levels of IL-21 resulted in the accumulation of immune complexes in the glomeruli, thickness of the glomerular basement membrane, and proteinuria (40).

To enhance our comprehension of the progression of SLE and LN, we analyzed the correlation between the miRNAs under investigation and cytokines. We observed a significant association between IL-21 and both IL-12 and miR-124. Additionally, we found that miR-199a was significantly correlated to miR-124, miR-146a and miR-21. Furthermore, there was a notable association observed between miR-146a and miR-21. These findings demonstrate that they are influenced by each other and point out the possible role of the studied miRNAs and interleukins in the development of SLE and LN.

The ROC analysis revealed that miR-199a and miR-146a exhibited the most noteworthy discriminatory ability, followed by miR-21, and to a lesser extent, miR-124, in discriminating between LN and SLE patients without LN. Additionally, miR-146a and miR-124 demonstrated additional discriminatory power in distinguishing SLE patients from the control group. On the other hand, logistic regression analysis revealed that only miR-199 could be used as LN predictor, thus, it can be used as potential predictive biomarker for LN.

The clinical course of SLE can impact various organs, including the joints, skin, kidneys, lungs, heart, vascular system, and brain. SLE patients exhibit a high prevalence of metabolic syndrome, which increase the risk of disease of cardiovascular system and type II diabetes (79). In our study, we investigated correlations between the studied parameters and certain clinical characteristics of LN. We observed that patients with metabolic syndrome had significantly elevated FC of miR-199, while those with hypertension showed increased FC of miR-146a, miR-21, and miR-199. Cases with dyslipidemia exhibited significantly elevated levels of IL-12 and increased FC of miR-124. These results underscore the probable involvement of these miRNAs in inflammation and the pathogenesis of the disease. In line with our findings, previous research shows that miR-124 was highly associated with lipid and glucose metabolism (80), nevertheless, miR-146 was downregulated in obesity (81).

Additionally, we observed lower FC of miR-146a in patients with pulmonary manifestations, miR-21 in patients with GIT involvement, IL-21 and miR-124 in those with vasculitis, and IL-12 and miR-124 in those with thrombosis. To our knowledge, just a few studies have characterized miRNA’s association with these clinical characteristics. Lopez-Pedrera et al. (82) demonstrated that miRNAs play a role in various pathological processes implicated in the atherogenic process. They highlighted that miR-146 was associated with endothelial dysfunction as well as inflammatory cytokines secretion, while both miR-146 and miR-21 are associated with monocytes recruitment, differentiation and activation (83, 84). Furthermore, miR-199 has been shown to be involved in oxidative stress (85). It was reported that aberrant expression of several miRNAs in SLE patients has been associated with a pro-oxidative state and mitochondrial dysfunction, which in turn contributes to inflammation and cardiovascular complications (86). Nevertheless, additional investigations are needed to assess the relevance of these parameters in these situations.

In our analysis of miRNA correlations with clinical and serological parameters, we found that miR-146a, miR-199a, and miR-21 are the most closely related to LN parameters. Specifically, all three were significantly correlated with 24-hour proteinuria, while miR-146a and miR-199a were also correlated with creatinine levels and SLEDAI score. Moreover, miR-199a showed correlations with urea levels, and miR-21 exhibited a correlation with nephritis duration, underscoring their inflammatory involvement in LN pathogenesis. Despite C3 and C4 levels being pivotal biomarkers for LN (21) only miR-21 showed a negative correlation with C4. Consistent with our findings, prior studies has observed that miR-21 is positively associated with 24-hour proteinuria (27) and negatively with C4 (63), whereas miR-146a has been correlated with proteinuria, SLEDAI score, and creatinine levels in separate studies (63, 87). However these previous studies did not perform regression analysis. In our study on performing regression analysis of all the correlated parameters with studied miRNAs and cytokines, none remained significant. Only the association of serum creatinine and SLEDAI with miR146 remained significant.

Despite the negative correlation between miR-124 and the activity index, which supports its regulatory function in LN, it exhibited negative correlation with hemoglobin levels and positive correlation with creatinine levels. Given that, in our work, miR-124 levels were elevated in LN patients compared with SLE patients without LN, this might suggest a potential role for this miRNA in LN pathophysiology. Considering the scarcity of studies exploring the role of miR-124 in LN, further research is warranted to delineate its precise role in LN pathogenesis.

There is promising evidence that although there are no defined protocols for employing miRNAs in current clinical settings, they constitute a dependable tool for future use. These molecules fulfill the majority of the necessary criteria for being an optimal biomarker, including accessibility, being highly specific, and sensitive (88). Because of their unique characteristics such as being well-tolerated by the immune system and its ability to penetrate cell membranes easily, miRNAs show promising potential as a treatment option (19). Currently, there is limited research exploring the use of miRNA as a therapy for autoimmune diseases like SLE. Therefore, further studies aim to uncover the exact ways miRNA works and which pathways it affects in SLE and LN are needed. These studies shall pave the path for research exploring the potential role of miRNAs and their antagonists for treating SLE and LN in animal models and subsequently in human clinical trials.

Our work is subjected to certain limitations like the limited number of participants and the lack of follow-up for patients which might lead to fluctuation in miRNA’s level. Further studies are required with larger number of participants and longer-term monitoring of patients to assess their clinical progression and treatment outcomes.

## 5 Conclusion

In summary, our findings indicate the presence of a distinct miRNA signature in the sera of patients with LN. We found miR-199a, miR-21 and miR-146a to be the highest expressed in LN serum. We identified miR-199a as the most important LN predictor as ROC and logistic regression analysis exhibited its ability to discriminate LN patients from SLE patients without LN. MiR-146 was associated with creatinine level as well as disease activity as estimated by SLEDAI score, thus, it might have a role in disease pathogenesis.

## Data Availability

The raw data supporting the conclusions of this article will be made available by the authors, without undue reservation.
